# Early diagnostic suggestions improve accuracy of GPs: a randomised controlled trial using computer-simulated patients

**DOI:** 10.3399/bjgp15X683161

**Published:** 2014-12-29

**Authors:** Olga Kostopoulou, Andrea Rosen, Thomas Round, Ellen Wright, Abdel Douiri, Brendan Delaney

**Affiliations:** Department of Primary Care and Public Health Sciences, School of Medicine, King’s College London, UK.; Department of Primary Care and Public Health Sciences, School of Medicine, King’s College London, UK.; Department of Primary Care and Public Health Sciences, School of Medicine, King’s College London, UK.; Department of Primary Care and Public Health Sciences, School of Medicine, King’s College London, UK.; Department of Primary Care and Public Health Sciences, School of Medicine, King’s College London, UK.; Department of Primary Care and Public Health Sciences, School of Medicine, King’s College London, UK.

**Keywords:** clinical decision support systems, decision making, diagnosis, diagnostic errors

## Abstract

**Background:**

Designers of computerised diagnostic support systems (CDSSs) expect physicians to notice when they need advice and enter into the CDSS all information that they have gathered about the patient. The poor use of CDSSs and the tendency not to follow advice once a leading diagnosis emerges would question this expectation.

**Aim:**

To determine whether providing GPs with diagnoses to consider before they start testing hypotheses improves accuracy.

**Design and setting:**

Mixed factorial design, where 297 GPs diagnosed nine patient cases, differing in difficulty, in one of three experimental conditions: control, early support, or late support.

**Method:**

Data were collected over the internet. After reading some initial information about the patient and the reason for encounter, GPs requested further information for diagnosis and management. Those receiving early support were shown a list of possible diagnoses before gathering further information. In late support, GPs first gave a diagnosis and were then shown which other diagnoses they could still not discount.

**Results:**

Early support significantly improved diagnostic accuracy over control (odds ratio [OR] 1.31; 95% confidence interval [95%CI] = 1.03 to 1.66, *P* = 0.027), while late support did not (OR 1.10; 95% CI = 0.88 to 1.37). An absolute improvement of 6% with early support was obtained. There was no significant interaction with case difficulty and no effect of GP experience on accuracy. No differences in information search were detected between experimental conditions.

**Conclusion:**

Reminding GPs of diagnoses to consider before they start testing hypotheses can improve diagnostic accuracy irrespective of case difficulty, without lengthening information search.

## INTRODUCTION

Computerised systems for disease management, preventive care, and prescribing are used extensively in clinical practice.^1^ Computerised diagnostic support systems (CDSSs) have not enjoyed similar success over more than four decades of development,^2^ despite diagnostic error affecting large numbers of patients,^3^ and being the commonest cause of litigation against general physicians.^4^,^5^

The basic operation of the typical CDSS has remained the same throughout its history: the physician collects information about the patient, enters the information into the CDSS, and receives diagnostic suggestions. There are at least two problems with this approach. First, it requires that the physician decide to consult the system. Physicians, however, do not necessarily know when advice would help.^6^ In a naturalistic trial of Isabel, a physician-triggered CDSS, junior doctors in paediatric ambulatory care sought and examined the system’s advice only around 2% of the time.^7^

The second problem is that system advice comes late in the diagnostic process. Physicians are known to generate few diagnostic hypotheses at the start of the encounter (within seconds), which determine what information they will gather and how they will interpret it.^8^–^11^ Consequently, advice given late in the consultation, after a fair amount of information has been gathered, may be less effective in two ways. First, the information a physician will enter into the CDSS and its resulting advice may be biased by the hypotheses entertained.^12^ Physicians may omit checking important information or may normalise abnormal information that does not fit with their hypothesis.^10^ Second, once physicians have mentally represented the problem in a specific way and considered a potential cause, a cognitive set may develop,^13^,^14^ making them less open to the system’s suggestions. Therefore, a potentially more successful approach would be to present diagnostic suggestions as early as possible in the consultation, before physicians have started testing any diagnostic hypotheses. Such early suggestions could be triggered automatically, based on the reason for encounter (RfE) and information in the patient’s record.

To test whether providing physicians with hypotheses early in the process improves diagnostic accuracy, detailed patient cases were constructed and presented to GPs to diagnose and manage via a web tool, while on the phone with a researcher. There is evidence that such simulations provide a valid measure of the quality of clinical practice.^15^ The performance of GPs who received early diagnostic suggestions was compared with that of an unaided group of GPs (control). To reflect the current approach to diagnostic support, a group of GPs was also included who received diagnostic suggestions late in the process, based on the information each GP had gathered.

**How this fits in**

Currently, in order to use computerised diagnostic support systems (CDSSs), physicians are expected to recognise when they need advice, input all information that they have gathered about the patient into the system, and follow its advice, while they may have already settled on a diagnosis. This study shows that providing GPs with diagnoses to consider before they start gathering any information, based only on patient information from the record (age, sex, risk factors, and past medical history) and the current reason for encounter, can improve diagnostic accuracy, irrespective of case difficulty and GP experience. The improvement obtained in this study that used a fairly simple manipulation compares favourably with other studies that tested fully developed CDSSs.

## METHOD

### Materials

Chest pain, abdominal pain, and dyspnoea are common reasons for consulting GPs, and can be caused by a variety of conditions, some serious. Using a series of evidence-based reviews, nine patient cases were developed, three for each RfE. Each case contained background information about the patient, the RfE, and an exhaustive list of positive and negative symptoms and signs. The complete case information always allowed for a single correct diagnosis. In each case, a more common diagnosis could explain some of the patient’s symptoms (Box 1). Easy and more difficult cases were constructed to determine the effect of diagnostic support on a range of difficulty. To determine difficulty, a previous scheme by one of the authors was adapted.^16^

Box 1. **The correct diagnosis (underlined) and the main competing diagnosis for each patient case**

**Table:** 

**RfE**	**Main competing diagnoses**		
**Chest pain**	Angina versus musculoskeletal pain	Pulmonary embolism versus lower respiratory tract infection	Tuberculosis versus lower respiratory tract infection
**Abdominal pain**	Crohn’s disease versus enteritis	Appendicitis versus UTI	Ovarian cancer versus IBS
**Dyspnoea**	Childhood asthma versus bronchitis	Cor pulmonale versus COPD exacerbation	COPD and aortic stenosis versus COPD alone

COPD = chronic obstructive pulmonary disorder. IBS = irritable bowel syndrome. RfE = reason for encounter. UTI = urinary tract infection.

To determine the relevant diagnoses for each case accurately and ensure completeness, diagnostic suggestions were adapted from DXplain, a stand-alone CDSS designed for general internal medicine (http://dxplain.org). The background information about each patient (age, sex, risk factors, current medications, and past medical history) and the RfE (chest pain, abdominal pain, or dyspnoea) were entered into DXplain. DXplain then delivered a list of suggested diagnoses, which was scrutinised by two authors who were GPs to ensure its appropriateness for UK primary care. The average list length was 17 diagnoses (range 9–22), and the correct diagnosis was always present. These diagnostic lists were used as such in early support and formed the basis for late support.

Late support was individualised, taking into account the information that a GP had gathered. It consisted of a list of diagnoses that could still not be discounted at the end of the GPs information search. These diagnoses were a subset of the respective full list described above, to which predetermined exclusion rules were applied, formulated via clinical consensus. The rules determined the diagnoses that could be reasonably discounted from the full list, had a GP asked specific questions. For example, for the patient presenting with chest pain, if the GP had checked about chest wall tenderness (negative finding), it was assumed that costochondritis could be discounted.

### Sample size

Sample size was calculated based on data from a previous study where 84 GPs diagnosed seven challenging cases on the computer.^16^ Mean diagnostic accuracy (proportion of correct diagnoses over all diagnoses) was 0.42, representing the expected accuracy of the control group. An intra-cluster correlation coefficient of 0.057 suggested significant clustering of responses within GPs. A two-sample comparison of proportions to detect an 8% increase in accuracy (from 0.42 to 0.50) with a power of 0.80 would require 633 responses per comparison group. This was multiplied by 1.456 (the ‘design effect’) and divided by nine cases, which gave 102 GPs per group.^17^

### Participants

Practices across England were invited to participate via the National Institute for Health Research Clinical Research Network.^18^ Their GPs could contact the study team, if they wished to participate. GPs were offered funding at standard clinical rates for an estimated 3-hour involvement, and individualised feedback, which they could use towards continuing professional development requirements.

### Procedure

Participants saw the nine cases in random order, in one of three experimental conditions: control, early support, or late support. Assignment to experimental conditions followed a predetermined blocked randomisation sequence that ensured equal numbers of participants per condition.

**Figure 1.**
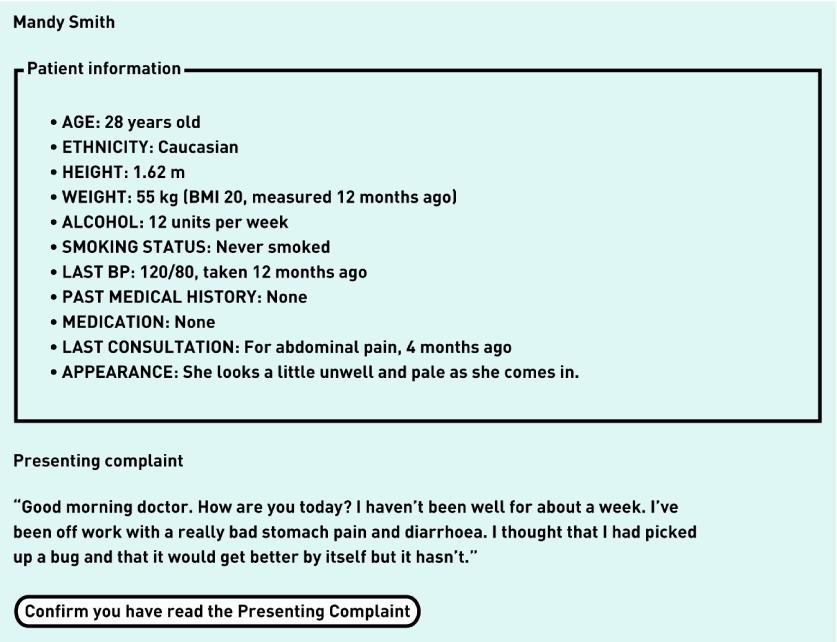
***The initial information that all GPs saw: example from a computer-simulated patient case.***

Data collection took place remotely over the internet using a web-tool designed specifically for the study. Participants were in simultaneous phone communication with a researcher (one of the authors) who operated the site and guided them through the task during a single session. After receiving training on one case, participants proceeded to diagnose and manage the nine cases. At the start of each case, all GPs read the initial information about the computer-simulated patient and the RfE (Figure 1). They could then request more information in relation to history, physical examination, and investigations. After each question, the researcher chose the appropriate answer from a predetermined list, and this was displayed on the GP’s screen. If participants asked questions for which there was no predetermined answer, the researcher selected appropriately from a set of generic responses, such as ‘no’ or ‘normal’. When participants wished to finish the consultation, they entered the diagnosis that they considered most likely and selected their management decision from a list of options (refer, prescribe, arrange follow-up, give advice, or wait and see). They then continued with the next patient. The system automatically recorded all information requests in sequence, the timing of each request, the diagnoses, and the management decisions.

**Figure 2.**
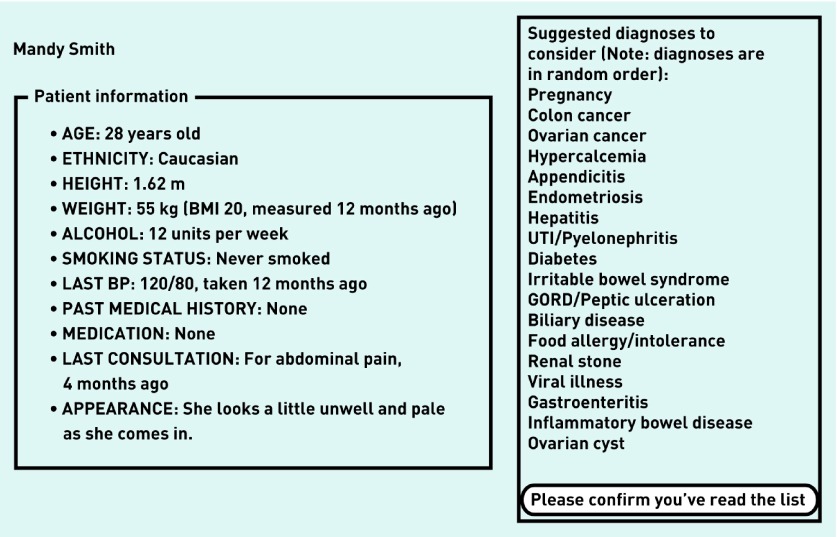
***The list of suggestions seen by the early support group: example of a computer-simulated patient case.***

This was the procedure for the control group. The early support group followed the same procedure with one important difference. After participants confirmed that they had read the initial information about the patient and the RfE, they were presented with a list of diagnostic suggestions (Figure 2). These suggestions were presented in random order for each participant. The list remained on the screen for a minimum of 20 seconds. In order to proceed, participants confirmed that they had read it. The list disappeared and they could start asking questions about the patient. They could recall the list at any time by pressing a button on the screen.

**Figure 3.**
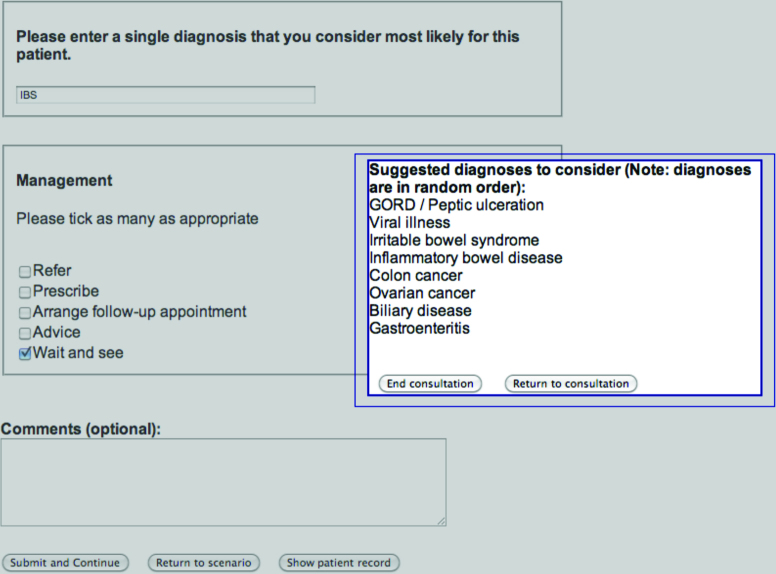
***Example screen with the list of suggestions seen by the late support group, after entering a diagnosis and management.***

GPs in the late support group proceeded in the same way as the control group, until they submitted a preliminary diagnosis and management, which triggered the list of diagnostic suggestions, presented in random order (Figure 3). GPs could then choose to ask more questions about the patient and/or change their diagnosis and management if they wished.

### Analyses

Diagnosis was scored as correct/incorrect and management as appropriate/ inappropriate, based on whether patient harm could result from either failing or delaying to deal with the condition. The effect of experimental condition on diagnostic accuracy was measured using mixed-effects logistic regression. Case difficulty (low, moderate, or high) was included as a factor and GP experience as a covariate. Two interactions (condition with difficulty and condition with experience) were also included to determine whether the effect of condition differed by difficulty and experience. Results are first reported from a model with experimental condition as the only factor and then from the adjusted model, as recommended in the literature.^19^

The influence of experimental condition on information search (number of information requests and time taken) was explored using mixed-effects linear regression, and the influence of diagnostic accuracy on management was explored using mixed-effects logistic regression. All regression models used random intercept to account for clustered data within participants, and case as a repeated measure.^20^ Stata (version 13.1) was used to analyse the data.

## RESULTS

A total of 297 GPs were recruited, including 30 trainees to reflect the proportion of trainees in the UK GP population. The sample had an average number of 9 years in general practice (SD = 9, median 5, range 0–34) and contained more women (54%) than the UK average (44%).^21^

Mean diagnostic accuracy (proportion of correct diagnoses over all diagnoses) was 0.63 for control (95% confidence interval [95% CI] = 0.60 to 0.67), 0.69 for early support (95% CI = 0.66 to 0.73), and 0.65 for late support (95% = CI 0.62 to 0.70). There was a reliable effect of experimental condition on accuracy: the odds of diagnosing correctly were 1.31 times higher with early support than control (odds ratio [OR] 1.31; 95% CI = 1.03 to 1.66, *P* = 0.027). No reliable difference was detected between control and late support (OR 1.10; 95% CI = 0.88 to 1.37). When difficulty, experience, and the interactions were included in the model, the effect of early support almost doubled (OR 1.91; 95% CI = 1.13 to 3.21, *P* = 0.015). Cases of moderate and high difficulty were both diagnosed less accurately than easy cases (OR 0.43; 95% CI = 0.31 to 0.59, and OR 0.20; 95% CI = 0.14 to 0.28, respectively). No effect of experience (*P* = 0.41) and no significant interactions were detected. Neither was an effect of experimental condition on information search detected. Appropriateness of management was strongly associated with diagnostic accuracy (OR 52; 95% CI = 41.81 to 65.61, *P*<0.001).

A control risk of misdiagnosis of 0.37 (1.0 – 0.63) and an odds ratio of misdiagnosis with early support of 0.77 (95% CI = 0.60 to 0.97) gives a number needed to treat of 17 (95% CI = 9 to 146).^22^ This means that one patient in 17, of similar difficulty as the cases used and who would otherwise have been misdiagnosed, would be correctly diagnosed with early support. If the odds ratio from the full regression model is used, the number needed to treat is 7 (95% CI = 5 to 35).

## DISCUSSION

### Summary

This randomised controlled study establishes a priority for the design of diagnostic support for general practice in situations where misdiagnoses are likely, for example, when strong diagnostic features are absent or a more common disease could explain some of the symptoms. This priority is the need to intervene early before GPs start gathering information to test hypotheses. The study obtained a statistically significant improvement in the diagnostic accuracy of GPs by reminding them of possible diagnoses to consider early on in their encounters with a series of computer-simulated patients.

The study detected no effect of experience on diagnostic accuracy. This is consistent with other studies in general and emergency medicine, which found either no relationship or a negative relationship.^16^,^23^–^25^

### Strengths and limitations

The concept of the study is novel and its randomised controlled design provides an assurance of the robustness of the findings. Most studies have evaluated the performance of specific CDSSs (whether they generate the correct diagnosis),^26^,^27^ rather than the performance of physicians using them.^7^,^25^,^28^ Furthermore, randomised designs in CDSS evaluation studies are rare.^29^

Studies evaluating the impact of a CDSS on physician accuracy use exclusively difficult cases. This study used cases ranging in difficulty to determine the potential effectiveness of diagnostic support on a more representative sample of GPs’ workload. The easy cases included strongly diagnostic features and the competing diagnoses had few overlapping features. As a result, they were diagnosed accurately more frequently than the other cases. The lack of a significant interaction between experimental condition and difficulty suggests that early support can improve accuracy across a wide range of difficulty. Furthermore, it can do so without significantly increasing time or the amount of information gathered. However it should, be acknowledged that even in the easy cases, the correct diagnosis was less common than the main competitor (Box 1). These are indeed the situations where, once a conclusion is reached prematurely, it may lead to misdiagnosis. Thus, they are the type of situations that could benefit from diagnostic support, and are typical of the case mix of diagnostic error in primary care.^30^

Although the study did not test a specific CDSS, some design decisions still had to be made in order to deliver the diagnostic support. Therefore, the results are tied to these decisions and may not generalise to systems that do not adopt them. For example, the early list of diagnostic suggestions remained on screen for at least 20 seconds and participants had to confirm that they read it before proceeding. This was done to ensure that the list was read. Furthermore, the choice was made not to present diagnoses in order of prevalence but to randomise the order for each participant, given that diagnoses appearing low on a list might be ignored. In short, support was designed with the principle to be tested in mind, rather than a future CDSS.

### Comparison with existing literature

Evaluation studies of CDSSs, measuring accuracy in a comparable way to the current study, produced more modest improvements. In an evaluation of two CDSSs, Iliad and QMR, 144 general internists diagnosed nine difficult cases first without and then with either CDSS.^12^ Participants were asked to generate a list of up to six diagnostic hypotheses for each case. Responses were considered accurate, if the correct diagnosis was included in the list. Mean accuracy increased from 46.4% at baseline to 50.8% with CDSS use; an absolute increase of 4.4% (with data omitted from 24 medical students). In another study that evaluated the effectiveness of Isabel, 39 internal medicine physicians diagnosed 12 cases on computer, first unaided and then using Isabel.^25^ The outcome measure was ‘errors of omission’, that is, failure to include all clinically important diagnoses as determined by two experts. Physicians made on average 5.06 errors of omission unaided and 4.61 errors of omission with the CDSS; a reduction of 0.44 (with data omitted from 13 medical students). Although avoiding an omission error will not necessarily result in the correct diagnosis, it may improve diagnostic accuracy. Thus, the 6% improvement that was obtained with the simple manipulation in the current study compares favourably with fully developed CDSSs.

### Implications for research

Decision support delivered via the electronic health record (EHR] has the potential to improve the quality and safety of patient care.^2^ This study sends a promising message that capturing the RfE and using it to trigger and deliver diagnostic suggestions early and from within the patient’s EHR could alone reduce diagnostic error and therefore deserves further development into a CDSS. The authors have now developed a diagnostic tool prototype that relies on the principle of early support and integrates with the EHR. It is currently being evaluated with GPs consulting with standardised patients (actors).
